# ZetaHunter, a Reproducible Taxonomic Classification Tool for Tracking the Ecology of the *Zetaproteobacteria* and Other Poorly Resolved Taxa

**DOI:** 10.1128/MRA.00932-18

**Published:** 2018-08-23

**Authors:** Sean M. McAllister, Ryan M. Moore, Clara S. Chan

**Affiliations:** a School of Marine Science and Policy, University of Delaware, Newark, Delaware, USA; b Center for Bioinformatics and Computational Biology, University of Delaware, Newark, Delaware, USA; c Department of Geological Sciences, University of Delaware, Newark, Delaware, USA; Louisiana State University

## Abstract

Like many taxa, the *Zetaproteobacteria* lack well-defined taxonomic divisions, making it difficult to compare them between studies. We designed ZetaHunter to reproducibly assign 16S rRNA gene sequences to previously described operational taxonomic units (OTUs) based on a curated database. While ZetaHunter can use any given database, we included a curated classification of publicly available *Zetaproteobacteria*.

## Abstract (precis)

Like many taxa, the *Zetaproteobacteria* lack well-defined taxonomic divisions, making it difficult to compare them between studies. We designed ZetaHunter to reproducibly assign 16S rRNA gene sequences to previously described operational taxonomic units (OTUs) based on a curated database.

## ANNOUNCEMENT

Taxonomic groups with limited cultivated representatives are often lumped into a single taxonomic label encompassing multiple distinct ecological units. This makes it difficult to explicitly discuss the abundance, significance, and niche preference of these units between studies. One group with poor taxonomic resolution is the class *Zetaproteobacteria*, which is only accurately classified to this taxonomic level by standard small subunit rRNA (16S rRNA) gene classification tools. ZetaHunter allows for reproducible, higher-resolution comparisons across studies by using a curated data set with a defined taxonomy based on operational taxonomic units (OTUs). ZetaHunter comes with a curated database to identify the members of the *Zetaproteobacteria*, though it may be used with any curated 16S rRNA gene database. (This article was submitted to an online preprint archive [1].)

ZetaHunter is a command line program written in Ruby designed to assign user-supplied 16S rRNA gene sequences to OTUs defined by a reference sequence database. ZetaHunter can be used on Linux, Mac OSX, and Windows platforms through a Docker container or through installation from source (Linux and Mac OSX only). By default, ZetaHunter uses a curated database of *Zetaproteobacteria* 16S rRNA genes from ARB SILVA (release 128) (2) and *Zetaproteobacteria* genomes from the Joint Genome Institute (JGI) Integrated Microbial Genomes (IMG) database (3). *Zetaproteobacteria* OTU (ZOTU) definitions include those reported by McAllister et al. (4) at 97% identity, maintaining ZOTU number order from ZOTU1 to ZOTU28 for ease in comparisons across studies. Numbered ZOTUs from ZOTU29 upward were discovered after 2011.

**FIG 1:**
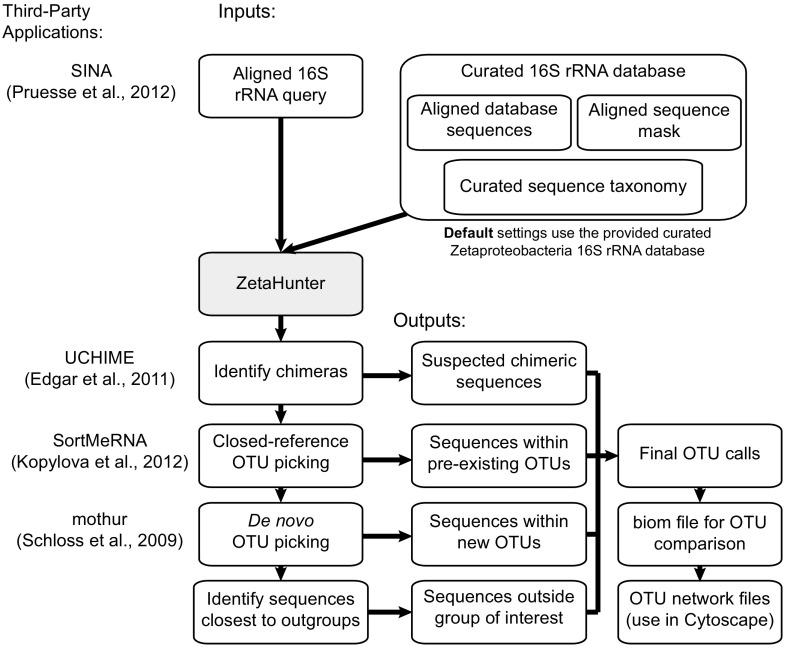
Flow chart showing the ZetaHunter pipeline. Third-party tools used in the pipeline are indicated to the left.

Input FASTA sequences for use in ZetaHunter must be aligned first using SINA (either online or standalone) (5). The default pipeline of ZetaHunter (Fig. 1) takes these SINA-aligned 16S rRNA gene sequences and processes them as follows: (i) input sequences are masked to the 1,282 bp used by McAllister et al. (4); (ii) sequences are checked for chimeras using the mothur UCHIME algorithm (6, 7); (iii) SortMeRNA is used to cluster new sequences with the reference database, assigning a ZOTU based on genetic distance (closed reference binning) (8); (iv) the remaining sequences are clustered into novel OTUs (NewZetaOtus) with mothur (*de novo* binning, average neighbor, numbered by abundance) (6); and (v) summary files (including final ZOTU calls and closest database hits), a biological observation matrix (biom) table (9) showing counts for each ZOTU by sample, and OTU network files are exported for use. The ZetaHunter database includes 21 proteobacterial outgroup sequences, allowing ZetaHunter to flag sequences potentially outside the *Zetaproteobacteria*. Additionally, sequences that are short, chimeric, or singletons/doubletons or contain ambiguous bases are also flagged. This pipeline is an implementation of open-reference OTU picking similar to the one found in QIIME (10).

To use ZetaHunter for non-*Zetaproteobacteria*, users need (i) a database of SINA-aligned 16S rRNA gene sequences, (ii) a sequence mask with asterisks at each informative alignment column to be used for taxonomic assignment, and (iii) a tab-delimited file assigning each sequence to a particular taxonomic group at the similarity threshold desired by the user.

### Data availability.

ZetaHunter is available for download at (https://github.com/mooreryan/ZetaHunter) (11), where it will be supported and maintained for at least the next 10 years. GitHub documentation includes installation instructions, a description of all dependencies, details of the ZetaHunter curated database, descriptions of output files, and examples of classifications of *Zetaproteobacteria* and non-*Zetaproteobacteria*. Detailed example data sets are included in the ZetaHunter_examples GitHub repository (https://github.com/mooreryan/ZetaHunter_examples).
